# Insulin deficiency exacerbates cerebral amyloidosis and behavioral deficits in an Alzheimer transgenic mouse model

**DOI:** 10.1186/1750-1326-5-46

**Published:** 2010-11-02

**Authors:** Xu Wang, Wei Zheng, Jing-Wei Xie, Tao Wang, Si-Ling Wang, Wei-Ping Teng, Zhan-You Wang

**Affiliations:** 1 Key Laboratory of Medical Cell Biology of Ministry of Education, and Key Laboratory of Endocrine Diseases of Liaoning Province, China Medical University, Shenyang, PR China; 2 Department of Histology and Embryology, Liaoning University of Traditional Chinese Medicine, Shenyang, PR China; 3 Department of Pharmaceutics, School of Pharmacy, Shenyang Pharmaceutical University, PR China

## Abstract

**Background:**

Although increasing evidence has indicated that brain insulin dysfunction is a risk factor for Alzheimer disease (AD), the underlying mechanisms by which insulin deficiency may impact the development of AD are still obscure. Using a streptozotocin (STZ)-induced insulin deficient diabetic AD transgenic mouse model, we evaluated the effect of insulin deficiency on AD-like behavior and neuropathology.

**Results:**

Our data showed that administration of STZ increased the level of blood glucose and reduced the level of serum insulin, and further decreased the phosphorylation levels of insulin receptors, and increased the activities of glycogen synthase kinase-3α/β and c-Jun N-terminal kinase in the APP/PS1 mouse brain. We further showed that STZ treatment promoted the processing of amyloid-β (Aβ) precursor protein resulting in increased Aβ generation, neuritic plaque formation, and spatial memory deficits in transgenic mice.

**Conclusions:**

Our present data indicate that there is a close link between insulin deficient diabetes and cerebral amyloidosis in the pathogenesis of AD.

## Background

Alzheimer's disease (AD) is a neurodegenerative disease clinically characterized by progressive cognitive impairment, and pathologically characterized by the presence of extracellular senile plaques and intracellular neurofibrillary tangles (NFTs) in the brain. Senile plaques are largely composed of amyloid-β (Aβ), which is a 4 kDa peptide derived from the amyloid-β precursor protein (APP). Processing of APP involves two major pathways, one non-amyloidogenic and one amyloidogenic. The non-amyloidogenic pathway is mediated by α- and γ-secretases and gives rise to a large fragment known as soluble APPα (sAPPα) and a small 3 kDa peptide p3. On the other hand, the amyloidogenic pathway is mediated by β- and γ-secretases and produces soluble APPβ (sAPPβ) and Aβ [1-3]. Aβ is toxic to neurons, and the deposition of Aβ and subsequent formation of senile plaques are considered to be the primary cause of AD [4].

The pancreatic β-cell-secreted hormone, insulin, predominantly acts by reducing blood sugar levels. Many studies have shown that both insulin and its target, insulin receptors (IR), are abundantly distributed throughout the brain and are involved in the regulation of glucose metabolism, food intake, and body weight [5]. Insulin also affects numerous brain functions including cognition and memory through complex insulin/IR signaling pathways [6]. Importantly, increasing evidence has indicated that brain insulin dysfunction is related to late onset AD [7]. First, AD patients present with reduced CSF insulin levels and impaired insulin-like signal transduction, compared with age-matched controls [8,9]. Second, AD patients with lower insulin levels have lower cognitive skills than those with normal insulin levels [10]. Third, disruption of cerebral IR functions by intracerebroventricular (icv) injection of streptozotocin (STZ), leads to AD-like changes and progressive cognitive impairment in an AD transgenic mouse model [11]. By contrast, icv administration of insulin improves memory formation in a passive-avoidance task in rats [12]. Most interestingly, recent clinical evidence suggests that diabetic patients treated with insulin may not develop AD [13,14]. Intranasal administration of insulin may have beneficial effect on the cognitive function without the risk of peripheral hypoglycemia in human [15,16]. Administration of insulin and glucose enhances the memory of AD patients to a greater extent than injection of glucose alone [17]. Furthermore, depletion of insulin by administration of STZ causes markedly increased levels of phosphorylated tau (p-tau) in the mouse brain [18,19], and increased levels of Aβ in the mouse brain [20], suggesting that insulin deficiency is involved in tau phosphorylation and Aβ generation. However, the mechanisms responsible for the effects of insulin deficiency on AD pathogenesis remain largely unknown.

In the present study, we evaluated the effect of insulin deficiency on APP processing and Aβ generation using an APP/presenilin-1 (APP/PS1) double transgenic mouse model treated with STZ. Our results indicate that insulin deficiency reduces IR phosphorylation, promotes APP processing, accelerates cerebral amyloidosis and exacerbates spatial memory deficits in these transgenic mice. The present data underscore the potential role of insulin deficiency in the pathogenesis of AD.

## Results

### High blood glucose and low serum insulin levels in STZ-treated APP/PS1 mice

**Figure 1:**
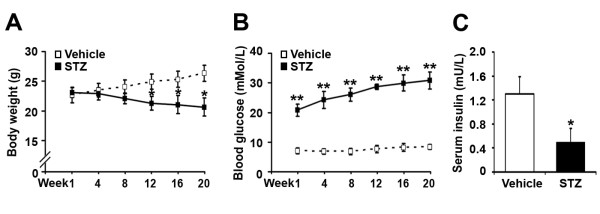
**The body weight, blood glucose and serum insulin levels in APP/PS1 mice**. APP/PS1 mice were injected intraperitoneally with STZ (90 mg/kg) for 2 consecutive days, while age-matched control APP/PS1 mice received citrate buffer only. (A) The changes in body weight in vehicle controls and STZ-induced diabetic APP/PS1 mice. From week 12 to 20 after STZ treatment, the body weights of STZ-treated mice were lower than those of the vehicle controls. (B) The levels of blood glucose in controls and STZ-treated APP/PS1 mice. From week 1 to 20, STZ treatment significantly increased the levels of blood glucose, compared with controls. (C) At the 20th week after STZ treatment, the serum insulin level was measured. A significant reduction in serum insulin levels could be detected in STZ-induced diabetic APP/PS1 mice, compared with vehicle control mice. *p < 0.05; **p < 0.01 (n = 7).

The body weight and blood glucose of the APP/PS1 mice were measured at week 1, 4, 8, 12, 16 and 20 after STZ administration. As time increased, the body weights were gradually decreased in STZ-treated mice, but increased in age-matched controls. STZ-treated mice had lower body weights from week 12 to 20, compared with vehicle controls (Figure 1A; p < 0.05). Blood glucose levels of STZ-treated mice gradually increased with time, but no significant changes were observed in the controls. STZ-treated mice had higher levels of blood glucose from week 1 to 20, compared with vehicle controls (Figure 1B; p < 0.01).

To determine whether STZ treatment reduced the pancreatic insulin secretion in APP/PS1 mice, the serum insulin levels were examined at the 20th week after STZ administration. The levels of insulin were 0.49 ± 0.24 mU/L in STZ-treated mice and 1.30 ± 0.29 mU/L in vehicle controls, respectively (Figure 1C). Statistical analysis showed that there was a 62% reduction in the serum insulin level of STZ-induced diabetic APP/PS1 mice, compared with vehicle controls (Figure 1C; p < 0.05).

### Insulin deficiency reduces insulin receptor phosphorylation in the APP/PS1 mouse brain

**Figure 2:**
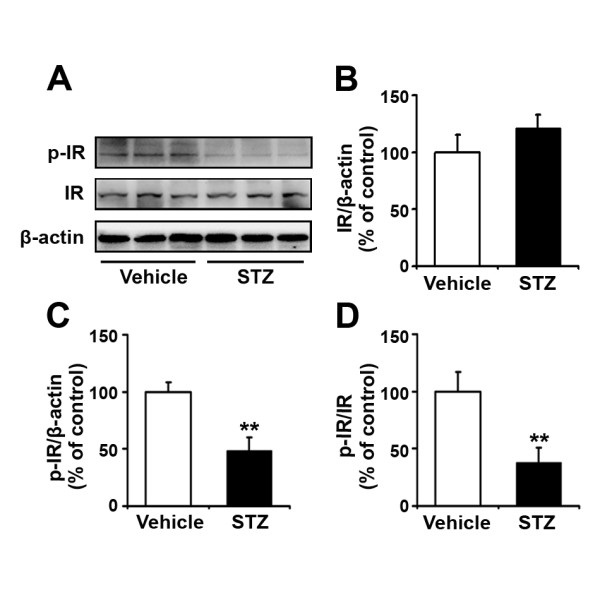
**Administration of STZ downregulates the phosphorylation levels of IR**. (A) Western blots showing the expression levels of phosphorylated insulin receptor (p-IR) and total IR proteins in APP/PS1 transgenic mouse brain 20 weeks after STZ administration. β-actin was used as a loading control. (B) There were no significant differences in total IR levels between vehicle controls and STZ-treated mice. (C) Insulin deficiency induced by STZ administration led to a marked reduction in the p-IR level in the transgenic mouse brain. (D) The ratio of p-IR/IR was significantly reduced in STZ-treated mouse brain, compared with the vehicle control. **p < 0.01 (n = 7).

Insulin binding to its receptor IR and its subsequent dimerization and autophosphorylation is the initial step in activation of the insulin/IR signaling pathways [21]. In the brain, the phosphorylation levels of IR represent the activity of IR protein. Thus, we examined the levels of both total IR protein and phosphorylated IR (p-IR), to evaluate the effects of insulin deficiency on IR activity in the STZ-induced diabetic APP/PS1 mouse brain. As shown in Figure 2, immunoblotting results showed that the level of total IR protein was not affected by STZ-induced insulin deficiency (Figure 2A and 2B; p > 0.05). However, the level of p-IR was significantly reduced by 52% in the STZ-treated mouse brain, compared with vehicle control (Figure 2A and 2C; p < 0.01). Further, the ratio of p-IR/IR was also markedly decreased by 63% in the STZ-treated mouse brain (Figure 2D; p < 0.01).

### Effects of insulin deficiency on GSK3, ERK and JNK phosphorylation in the APP/PS1 mouse brain

**Figure 3:**
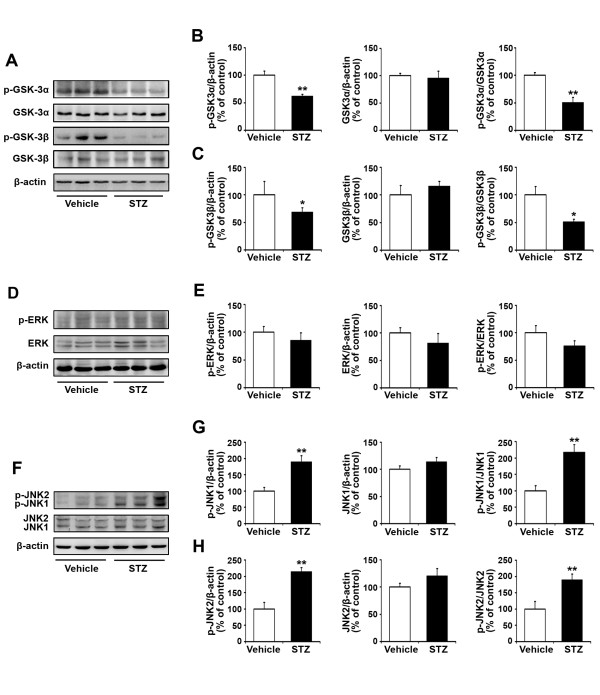
**Expression levels of GSK, ERK and JNK in the APP/PS1 mouse brain 20 weeks after STZ treatment**. (A) Immunoblot analyses showing the protein levels of GSK-3α and GSK-3β in transgenic mouse brain. β-actin was used as a loading control. (B) A marked reduction in p-GSK3α level was detected in STZ-treated mouse brain. Also the ratio of p-GSK3α/GSK3α was significantly reduced after STZ treatment. (C) Insulin deficiency reduced GSK3β phosphorylation, and resulted in a reduced ratio of p-GSK3β/GSK3β in STZ-induced diabetic mouse brain. (D) Immunoblot analyses showing the expression levels of ERK in transgenic mouse brain. β-actin was used as a loading control. (E) Administration of STZ resulted in a reduced ratio of p-ERK/ERK, but this was not statistically significant compared with the vehicle control. (F) Immunoblot analyses showing the expression levels of JNK in transgenic mouse brain. β-actin was used as a loading control. (G) STZ treatment increased JNK1 phosphorylation and the ratio of p-JNK1/JNK1 was significantly increased in STZ-induced diabetic mouse brain. (H) The expression levels of p-JNK2 and the ratio of p-JNK2/JNK2 were significantly increased in STZ-induced diabetic mouse brain. *p < 0.05, **p < 0.01 (n = 7).

To determine whether STZ treatment influences the activities of the downstream molecules in the IR-mediated signaling pathway, western blot analyses were employed to assess the expression levels of GSK3, ERK and JNK (Figure 3), which have been previously shown to be associated closely with Aβ generation and senile plaque formation [22-24]. The expression levels of both total proteins and phosphorylated forms of GSK3α, GSK3β, ERK and JNK were analyzed (Figure 3A, D and 3F). STZ treatment significantly reduced the level of p-GSK3α (Figure 3B; p < 0.01), whereas the total protein level remained unchanged (Figure 3B; p > 0.05). Thus, the ratio of p-GSK3α/GSK3α was significantly reduced by 50% in STZ-induced diabetic mice compared with controls (Figure 3B; p < 0.01). Similarly, the expression level of p-GSK3β was reduced, and the ratio of p-GSK3β/GSK3β was significantly reduced by 49% in STZ-induced diabetic mice (Figure 3C; p < 0.05). STZ treatment led to a decrease in the ratio of p-ERK/ERK by 24%, but this was not statistically significant, compared with the vehicle control (Figure 3E; p > 0.05). Furthermore, activation of the JNK signaling pathway was detected in the STZ-treated mouse brain, and the ratio of p-JNK1/JNK1 and p-JNK2/JNK2 was increased by 118% and 89%, respectively, compared with the vehicle control (Figure 3G and 3H; p < 0.01). Taken together, these data suggest that insulin deficiency reduces the phosphorylation of GSK3α/β, enhances the phosphorylation of JNK1/2, and, thus, increases GSK3 and JNK activities in STZ-treated APP/PS1 mouse brain.

### Insulin deficiency enhances APP processing in the APP/PS1 mouse brain

**Figure 4:**
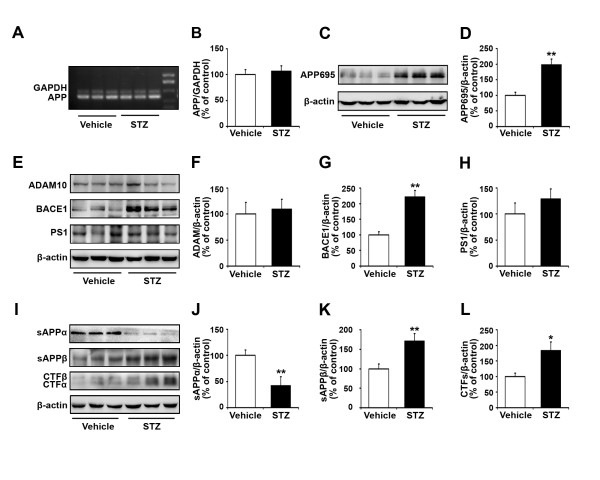
**Expression levels of APP, cleavage enzymes and fragments of APP in STZ-treated transgenic mouse brain**. (A) RT-PCR analysis was performed to determine the levels of APP mRNA in APP/PS1 transgenic mouse brain. GAPDH was used as an internal reference gene. (B) There were no significant differences in expression levels of APP mRNA between in the STZ- and vehicle-treated transgenic mouse brain. (C) Immunoblotting showing the expression levels of APP695 in vehicle control and STZ-treated APP/PS1 transgenic mouse brain. β-actin was used as a loading control. (D) Administration of STZ significantly increased the expression level of APP695 in the transgenic mouse brain. (E) Immunoblotting showing the expression levels of ADAM10, BACE1, PS1 in transgenic mouse brain. β-actin was used as a loading control. (F) STZ treatment did not affect the expression level of ADAM10 protein. (G) STZ-induced insulin deficiency resulted in a marked increase in BACE1 protein in the transgenic mouse brain. (H) STZ treatment resulted in an increased level of PS1 protein, but the difference was not statistically significant, compared with the vehicle control. (I) Immunoblotting showing the expression levels of sAPPα, sAPPβ and CTFs in transgenic mouse brain. β-actin was used as a loading control. (J) STZ-induced insulin deficiency significantly reduced the levels of sAPPα. (K) STZ treatment resulted in a markedly increased level of sAPPβ in transgenic mouse brain. (L) STZ treatment significantly increased the level of CTFs. *p < 0.05, **p < 0.01 (n = 7).

GSK3 and JNK have been known to affect Aβ generation by modulation on APP processing [25,26]. To verify the effects of STZ treatment on APP processing, we measured the expression levels of APP mRNA and APP695 protein in APP/PS1 mouse brain (Figure 4). RT-PCR results showed that there were no significant differences in levels of APP mRNA between STZ-treated mouse brain and control (Figure 4A and 4B; p > 0.05). However, immunoblot analysis revealed that STZ treatment increased the level of APP695 protein by 98%, compared with the control (Figure 4C and 4D; p < 0.01).

We then measured the activities of α-, β- and γ-secretase in response to insulin deficiency using antibodies against ADAM10, BACE1 and PS1, respectively (Figure 4E). The results obtained showed no detectable changes for the ADAM10 and PS1 (Figure 4F and 4H; p > 0.05). However, the level of BACE1 was significantly increased by 122% in the insulin-deficient mouse brain relative to the control (Figure 4G; p < 0.01).

Next, we measured the expression levels of APP cleavage fragments that are important to explain the production of Aβ, including sAPPα, sAPPβ and CTFs, in the brain samples of APP/PS1 mice (Figure 4I). Insulin deficiency reduced the expression level of sAPPα by 58% (Figure 4J; p < 0.01), and increased the levels of sAPPβ by 71% (Figure 4K; p < 0.01). Western blot analysis using an anti-CTFs antibody showed a significant 84% increase in the production of CTFs in STZ-induced diabetic mice relative to vehicle controls (Figure 4L; p < 0.05).

Taken together, these results suggest that insulin deficiency regulates the amyloidogenic processing of APP in the APP/PS1 transgenic mouse brain.

### Insulin deficiency accelerates Aβ plaque formation in APP/PS1 mice

**Figure 5:**
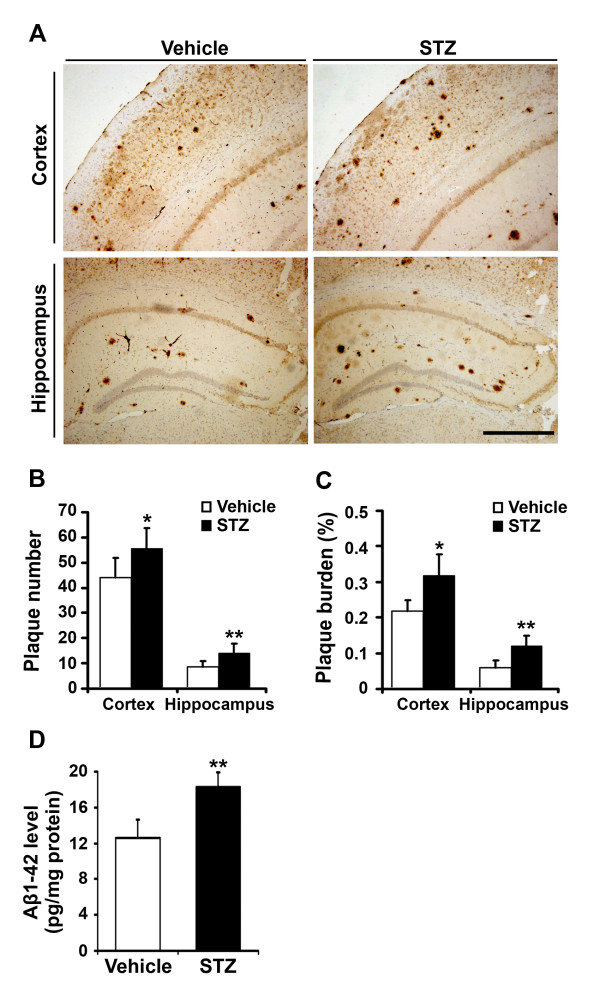
**STZ-induced insulin deficiency accelerates plaque formation and Aβ secretion in the APP/PS1 mouse brain**. (A) Twenty weeks after STZ administration, the senile plaques were detected using Aβ immunohistochemistry, and Aβ levels were measured by ELISA in APP/PS1 transgenic mouse brain. The representative images of Aβ-immunostained brain sections from vehicle control and STZ-treated transgenic mice, respectively. Note the number and size of Aβ-positive plaques were increased in the cortex and hippocampus in STZ-treated mice, compared with the control. Scale bar = 100 μm. (B) STZ-induced insulin deficiency significantly increased the number of plaques in the cortex and hippocampus in the transgenic mouse. (C) Insulin deficiency led to an increase in the plaque burden in the cortex and hippocampus of the mouse brain. (D) The level of soluble Aβ1-42 was measured using an ELISA kit. STZ treatment significantly increased the levels of Aβ1-42 peptide in APP/PS1 transgenic mouse brain. * p < 0.05, ** p < 0.01 (n = 7).

To investigate the effect of insulin deficiency on Aβ deposition and senile plaque formation, sections from vehicle- and STZ-treated APP/PS1 mouse brains were stained with an antibody against Aβ. In general, Aβ-immunoreactive plaques were distributed throughout the cortex and hippocampus in both vehicle- and STZ-treated APP/PS1 mouse brain. However, both the number and size of the Aβ-immunoreactive senile plaques were markedly increased in the cortex and hippocampus in the STZ-treated mouse brain (Figure 5A). Image analyses showed a significant effect of insulin deficiency on Aβ neuropathology. Quantitative analysis of the plaque numbers showed a 26% increase in the cortex and a 62% increase in the hippocampus, respectively (Figure 5B). Changes in Aβ burden were also analyzed by measuring the areas of neuritic plaques in vehicle- and STZ-treated APP/PS1 mouse brain. In STZ-treated mice, the area of Aβ plaques was significantly increased by 1.5-fold in the cortex and by 2-fold in the hippocampus, respectively (Figure 5C). Levels of Aβ1-42 in STZ-induced diabetic APP/PS1 mouse brains were also measured by Sandwich ELISA. Aβ1-42 levels were markedly increased in STZ-treated APP/PS1 mice relative to controls (12.63 ± 1.97 pg/mg vs. 18.30 ± 1.60 pg/mg, p < 0.01) (Figure 5D).

### Insulin deficiency exacerbates cognitive impairment in STZ-induced APP/PS1 diabetic mice

**Figure 6:**
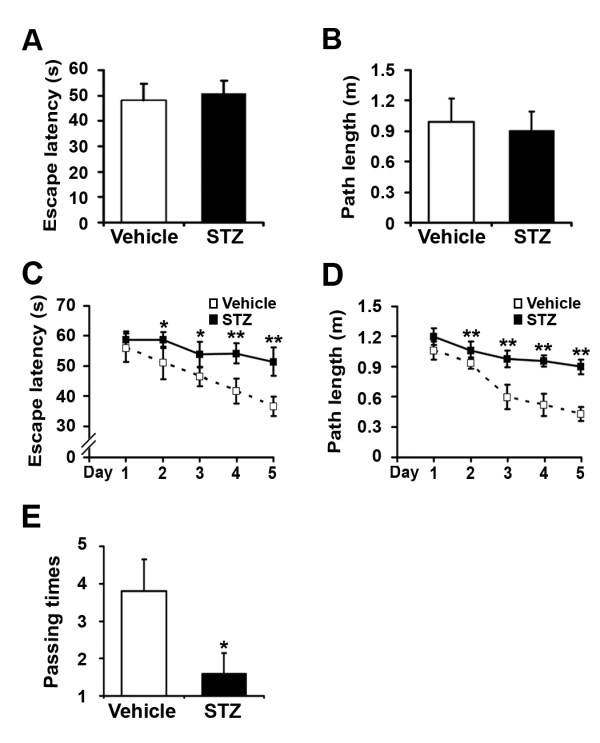
**STZ treatment exacerbates spatial learning impairment in APP/PS1 mice**. Morris water maze tests consisted of 2 days of visible platform training, 5 days of hidden platform testing, and a probe trial 24 h after the last hidden platform test. (A, B) The visible platform tests showed that no significant differences in escape latency and swimming distances were detected between vehicle and STZ-treated transgenic mice. (C, D) The hidden platform tests showed that STZ treatment significantly increased both the escape latency and swimming length from the 2nd to 5th day. (E) The probe trial test showed that administration of STZ significantly reduced the time that mice traveled into the center of the quadrant, where the hidden platform was previously located. *p < 0.05, **p < 0.01 (n = 7).

To investigate whether insulin deficiency affects learning and memory in STZ-treated APP/PS1 mice, behavioral tests were performed at 19 weeks after STZ treatment. In the visible platform test, we observed that STZ-induced diabetic mice and vehicle controls had a similar escape latency (50.65 ± 5.18 vs. 48.07 ± 6.65; Figure 6A; p > 0.05) and path length (0.99 ± 0.23 vs. 0.90 ± 0.19; Figure 6B; p > 0.05), indicating that STZ-induced insulin deficiency did not affect the motility or vision in our mouse model. In the place navigation (hidden platform) tests, STZ-treated APP/PS1 mice spent more time searching for the hidden platform than vehicle controls. From the 2nd to 5th day, the escape latencies of STZ-induced diabetic mice and controls were 58.62 ± 2.61 vs. 51.04 ± 5.34 (p < 0.05), 53.81 ± 4.15 vs. 46.44 ± 3.05 (p < 0.05), 54.26 ± 3.35 vs. 41.60 ± 4.16 (p < 0.01), 51.45 ± 4.67 vs. 36.60 ± 3.13 (p < 0.01), respectively (Figure 6C). The path length of STZ-treated mice and vehicle controls was 1.06 ± 0.09 vs. 0.94 ± 0.05 (p < 0.01), 0.98 ± 0.08 vs. 0.60 ± 0.12 (p < 0.01), 0.96 ± 0.05 vs. 0.62 ± 0.11 (p < 0.01), 0.92 ± 0.07 vs. 0.51 ± 0.07 (p < 0.01), respectively (Figure 6D). Moreover, in the probe trial on the last day of testing, the STZ-treated mice passed the former platform location significantly fewer times than the controls (1.60 ± 0.55 vs. 3.80 ± 0.84; Figure 6E; p < 0.05). Overall, these behavioral tests indicate that STZ-induced diabetic APP/PS1 mice show significant cognitive deficits in spatial learning and memory function.

## Discussion

The accumulating evidence that a lower expression of insulin and insulin receptors occurs in the brain of AD suggests a role of impaired insulin signaling pathway in the pathogenesis of AD [27,28]. Several epidemiological and experimental studies have demonstrated that impaired insulin and insulin signaling transduction occur in both diabetes mellitus and AD, suggesting a linkage between these two disorders [29-32]. In the present study, a mouse model of combined insulin-deficient diabetes and AD was made by injecting APP/PS1 transgenic mice with STZ, a known toxin for pancreatic insulin-producing β-cells. We found that administration of STZ markedly increased the level of blood glucose and reduced the level of serum insulin. Thus, we used this model to assess whether insulin deficiency exacerbated cerebral amyloidosis and behavioral deficits, and to explore the underlying mechanisms.

Consistent with previous reports, our findings showed that insulin deficient diabetes induced by STZ exacerbated the accumulation of Aβ in APP/PS1 transgenic mice [33]. First, a marked increase in APP695 suggests that the processing of APP by the non-amyloidogenic pathway is highly impaired, which is further confirmed by the very low levels of sAPPα. Since the non-amyloidogenic pathway is the major pathway for APP processing, an impaired α-cleavage explains the increased levels of APP without apparent increased transcription. Second, we observed that insulin deficiency enhanced the BACE1 expression and the sAPPβ fragment, resulting in increased soluble Aβ. Third, immunoblot analysis showed a significant increase in the C-terminal fragments of amyloid precursor protein (APP-CTFs) in STZ-induced diabetes. APP-CTFs are the substrates of γ-secretase and are also the direct precursor of Aβ [34,35]. Therefore, the increased expression levels of APP-CTFs could also be responsible for the Aβ accumulation.

Insulin is a polypeptide hormone consisting of a 21-amino acid α chain linked to a 30-amino acid β chain by two disulphide bonds [36]. It is synthesized primarily by β-cells of the pancreatic islets of Langerhans, and predominantly acts by reducing blood sugar levels in the periphery. In addition, insulin can be partially formed in the hippocampus, prefrontal cortex, entorhinal cortex, and the olfactory bulb [37]. In the brain, insulin exerts its pleiotropic effects through binding to its receptors and forms the insulin/IR complex [38], and triggers the IR to undergo dimerization and autophosphorylation [39]. The phosphorylated or active IR is involved in the activation of two major downstream signaling pathways, the phosphatidylinositol 3-kinase (PI3K) and mitogen-activated protein kinase (MAPK) pathways [20,40]. GSK3 is one of the key molecules downstream of the PI3K signaling pathway [41], and ERK and JNK are major components of two parallel cascades, respectively, in the MAPK pathways [42]. Importantly, GSK3, ERK and JNK have all been shown to be involved in the formation of pathomorphological AD hallmarks, such as Aβ plaques [43-45], hyperphosphorylated tau [46-50], and cerebral neuronal death [51-53].

Therefore, we examined the changes in activities of IR, GSK3, ERK and JNK in our mouse model. Our data showed that STZ-induced insulin deficiency reduces the phosphorylation levels of insulin receptors in the transgenic mouse brain. This is consistent with previous works showing that insulin deficiency inhibits the activity of its receptors and down-regulates the insulin/IR signaling pathway [20,33]. Further, we found that insulin deficiency enhances the activity of GSK3 and JNK, but not ERK. These findings suggest that STZ-induced insulin deficiency destroys insulin/IR signaling, abrogates the downstream GSK3 and JNK signaling pathways, and leads to the development of AD pathogenesis.

The activation of GSK3, including the α and β isoforms, through autophosphorylation has been implicated in AD pathogenesis [54]. Increased GSK3α activity is predominantly associated with the processing of APP and generation of Aβ [23], whereas activation of GSK3β is mainly involved in tau phosphorylation and the formation of NFTs [55-57]. Several studies have shown that GSK3 is closely linked to the mechanisms by which STZ-induced dysfunction of insulin cascades promotes the formation of Aβ plaques and NFTs [18,19,33]. Following an icv administration in the STZ-induced insulin-resistant brain state (IRBS) rat model, alterations in GSK3 proteins have been observed downstream of the PI3K pathway of the IR signaling cascade in the hippocampus [57]. Using a STZ-induced insulin deficiency APP/PS1 transgenic mouse model, we have shown for the first time that brain insulin deficiency leads to enhancement of GSK3α/β activation, increased cerebral amyloidosis, and exacerbation of behavioral deficits. Although the mechanism of GSK3 modulation in the AD brain is far from completely understood, it is reasonable to speculate that the regulation of insulin deficiency in APP processing to the amyloidosis pathway is, at least partly, through activation of GSK3 signaling.

JNK is a member of MAPK, also known as a stress-activated protein kinase, and the activation of JNK has been found in brains of AD patients [58,59]. In vitro studies have shown that intracellular Aβ accumulation triggers JNK activation, leading to neuronal death [60,61]. In Tg2576/PS1 double transgenic mice, JNK activation was associated with age-dependent Aβ deposition, tau phosphorylation, and the loss of synaptophysin [47,62]. Other reports have demonstrated that BACE1 activation is related to a mechanism involving JNK activation during oxidative stress [63,64]. Furthermore, JNK up-regulates phosphorylated Thr668, the main phosphorylation site of APP [65], and participates in the endoproteolytic cleavage of APP by BACE1 [66]. In our model mice, we showed that STZ-induced insulin deficiency promotes JNK activation in the transgenic mouse brain. Furthermore, insulin deficiency enhances the expression level of BACE1 protein. The present data suggest that there is a close link between the activation of JNK and BACE1 in the case of insulin deficiency-induced cerebral amyloidosis.

We demonstrated that insulin-deficient diabetes in APP/PS1 transgenic mice exacerbated cognitive deficits, characterized by excessive Aβ accumulation and subsequent formation of senile plaques. However, it could not be ruled out that STZ treatment itself leads to impaired cognitive performance via other mechanisms related to IR signaling. Previous studies have shown that lithium as a specific GSK3 inhibitor may prevent the cognitive deficits [67,68]. Thus, GSK3 has critical function for the induction of memory formation. Also, over-expression of GSK3β in mice prevents the induction of LTP [69] and causes a decrease in spatial learning [70]. In addition, insulin treatment could be essential for improving cognitive performance. Intranasal insulin could prevent cognitive decline and GSK3 activation in a mouse model of type I diabetes [71]. In addition, daily intranasal insulin treatment has been shown to lessen cognition impairment in patients diagnosed with early AD [72]. Collectively, this research suggests that insulin deficiency likely plays a role in the regulation of AD-associated cognitive deficits.

The present study provides evidence indicating that insulin deficiency is able to aggravate Aβ-associated neuropathology and memory deficits in APP/PS1 transgenic mouse model. Insulin deficiency could affect the pathogenesis of AD through mechanisms unrelated to Aβ metabolism. Interestingly, tau hyperphosphorylation at Thr231 and Ser396 sites was significantly increased in insulin-deficient APP/PS1 mice (data not shown). Tau hyperphosphorylation is a key event in the pathogenesis of AD. *In vivo *and *in vitro *studies have shown that insulin can affect tau phosphorylation through the activation of GSK3β [18,73]. In addition, the insulin-degrading enzyme (IDE) plays a critical role in the mechanism related to insulin deficiency and AD [74]. IDE has been shown to degrade not only insulin but also a number of other small proteins, including Aβ [75]. Therefore, insulin deficiency may also decrease IDE expression and cause reduced degradation of Aβ thus contribute to increase in Aβ in APP/PS1 mouse brain [33].

## Conclusions

Our current study demonstrates that insulin deficiency-induced impaired insulin signaling, including activation of its downstream GSK3α/β and JNK pathways, is related to the activation of the amyloidogenic APP processing resulting in increased generation and deposition of Aβ1-42 and spatial memory deficits in the APP/PS1 transgenic mouse brain. The present data indicate that dysfunction of the insulin signaling pathway during insulin deficient diabetes contributes to the enhanced progression of AD.

## Methods

### Animals and induction of insulin-deficient diabetes

APP/PS1 mice expressing a chimeric mouse/human APP (Mo/HuAPP695swe) and a mutant human presenilin 1 (PSEN1dE9) were obtained from the Jackson Laboratory (West Grove, PA, USA). The APP/PS1 mouse was selected because of the high levels of Aβ1-42 generation and neuritic plaque formation in the brain, and relatively early appearance of behavioral deficits in the water maze test.

Throughout the experiments, APP/PS1 mice were kept in cages in a controlled environment (22-25 °C, 50% relative humidity, 12 h light/dark cycle) with free access to food and water throughout the experimental period of 20 weeks. All animal experiments were performed in accordance with the care and use of medical laboratory animals (Ministry of Health PR China, 1998) and the permission of the laboratory animal ethical standards of China Medical University.

Three-month-old APP/PS1 mice were fasted for 12 h, and were intraperitoneally (i.p.) injected with STZ (90 mg/kg, Sigma, dissolved in sodium citrate buffer) for consecutive 2 days. Age-matched APP/PS1 mice received citrate buffer only (vehicle control). Mice with non-fasting blood glucose levels higher than 15 mM/L one week after STZ injection were considered to be diabetic and used in the later experiments. During the experimental period, 5 mice in the STZ-treated group died, probably due to the high blood glucose level and, finally, 7 mice in the STZ-treated group and 7 mice in the vehicle control group were used for subsequent experiments at 19-20 weeks after STZ or vehicle treatment.

The body weight and blood glucose levels of the mice were monitored for 20 weeks. Blood samples were obtained by tail prick and blood glucose levels were measured using a portable blood glucose meter (Johnson, m211667, USA). At the 20th week after STZ administration, blood was extracted from the heart and the serum insulin level was determined by an Automated Chemiluminescence System (Bayer, ADIVA Centaur, USA).

### Morris water maze

At the 19th week after STZ treatment, mice were given behavioral tests with a Morris water maze for consecutive 8 days, including visible platform training, a navigation test and probe trial, as previously described with few modifications [76]. In brief, mice were trained individually for 2 days (3 trials with an interval of 30 min) to find the visible escape platform. At the 3rd-7th day, the platform was placed just below the water surface for the place navigation test, and each mouse was subjected to 3 trials per day at an interval of 1 min. For each trial, the latency and the path length by which the mouse found the hidden platform were recorded. At the 8th day, the platform was removed from the water for the probe trial. The number of times that each mouse crossed the center of the quadrant (where the platform was previously located) at an interval of 1 min was recorded. Finally, data on the escape latency, the path length and the number of passing times for the STZ- and vehicle-treated mice were compared and analyzed statistically with a computer program (ZH0065, Zhenghua Bio-equipments, PR China).

### Tissue preparation

After behavioral tests, mice were given an overdose of phenobarbital, and the blood was extracted from the heart for serum insulin measurement. Then, the mice were sacrificed by decapitation, and the brains were rapidly removed and cut sagittally into left and right hemispheres on an ice-cooled board. The right hemisphere was fixed in 4% paraformaldehyde and routine paraffin sections (7 μm) were prepared for morphological analysis. The hippocampus and cerebral cortex were dissected from the left hemisphere and stored at -80°C for sandwich enzyme-linked immunosorbent assay (ELISA) and Western blot analyses.

### Immunohistochemistry and image analysis of plaque pathology

Aβ immunohistochemical staining was performed to analyze the distribution of Aβ plaques in the APP/PS1 mouse brain. Briefly, paraffin sections were dewaxed, rehydrated and treated in 0.1 M Tris-HCl buffer (TBS, pH 7.4) containing 3% hydrogen peroxide (H_2_O_2_) for 10 min. After washing with TBS, sections were boiled in citric acid buffer for 5 min in a microwave oven. The sections were then rinsed, treated with 5% bovine serum albumin for 30 min, and incubated overnight with mouse anti-Aβ antibody (1:500, Sigma) at 4°C. After rinsing, sections were incubated with biotinylated goat anti-mouse IgG (1:200) for 1 h, followed by streptavidin peroxidase for 1 h at room temperature. After rinsing, the sections were treated with 0.025% 3,3-diaminobenzidine plus 0.0033% H_2_O_2 _in TBS for 10 min. The sections were dehydrated, cleared, covered and examined under a light microscope equipped with a digital camera (Olympus; AX70U-Photo, Japan). Control sections were treated with identical solutions but without primary antibody.

To assess the plaque number and Aβ burden in the cortex and hippocampus, 5 sections with the same reference position were selected from each mouse (n = 7). The number of plaques and the percentage of the total area of Aβ-positive areas compared with the total area of the cortex and hippocampus were quantified (in square micrometers). The data were analyzed with the image analysis system (Image-Pro Plus 6.0).

### Western blotting

Brain samples were minced into small pieces and homogenized in lysis buffer containing 150 mM sodium chloride, 50 mM Tris-hydrochloride, 1% Nonidet P-40, 0.25% sodium deoxycholate, 0.1% SDS, 1 mM phenylme-thylsulfonyl fluoride (PMSF), 10 mg/ml leupeptin, 1 mM Na_3_VO_4_, and 1 mM NaF, and incubated overnight at 4°C. The homogenate was centrifuged at 12,000 rpm for 30 min and the supernatant was divided into aliquots and frozen at -80°C. The total protein extract (60 μg) was separated on SDS-polyacrylamide gels and then transferred onto polyvinylidene difluoride membranes (Millipore, CA). Nonspecific binding sites on the membrane were blocked by 5% bovine serum albumin in 0.1% TBS/Tween-20 (TBST) for 1 h. The membranes were then incubated overnight at 4°C in specific primary antibodies: rabbit anti-APP695 (1:2000, Chemicon), rabbit anti-ADAM10 (1:1000, Millipore), rabbit anti-BACE1 (1:1000, Sigma), rabbit anti-presenilin 1 (PS1) (1:800, Millipore), rabbit anti-C-terminal fragments of APP (CTFs) (1:1000, Sigma-Aldrich), mouse anti-sAPPα (1:500, Immuno-Biological Laboratories), mouse anti-sAPPβ (1:500, Immuno-Biological Laboratories), phospho-APP (Thr668) (1:500, Cell Signaling Technology), rabbit anti-phospho-IR (Tyr972) (1:500, Millipore), rabbit anti-IR (1: 500, Millipore), rabbit anti-phospho-GSK3α (Ser21) (1:1000, Cell Signaling Technology), rabbit anti-GSK3α (1:1000, Cell Signaling Technology), rabbit anti-phospho-GSK3β (Ser9) (1:1000, Cell Signaling Technology), rabbit anti-GSK3β (1:1000, Cell Signaling Technology), rabbit anti-phospho-ERK1/2 (Thr202/Tyr204) (1:1000, Cell Signaling Technology), rabbit anti-ERK 1/2 (1:1000, Cell Signaling Technology), rabbit anti-phospho-JNK (Thr183/Tyr185) (1:1000, Cell Signaling Technology), rabbit anti-JNK (1:1000, Cell Signaling Technology) and mouse β-actin (1:5000, Santa Cruz Biotechnology).

After washing with TBST, the membranes were incubated with horseradish peroxide-conjugated second antibody (1:5000; Santa Cruz Biotechnology) for 1 h at room temperature. Immunoreactive bands were visualized using the Super Signal West Pico Chemi-luminescent Substrate (Pierce Biotechnology, Rockford, IL) using Chem Doc XRS with Quantity One software (BioRad, USA). The bands were scanned and the intensities of the bands were measured using Image-pro Plus 6.0 analysis software.

### RT-PCR

Tissue homogenates of the cortex of APP/PS1 mice treated with STZ and vehicle controls were collected. Total RNA was isolated using Trizol reagent (Invitrogen) and the RNA concentration was determined by measuring the absorbance at 260 nm. The total RNA of each sample was first reverse-transcribed into cDNA using EasyScriptTM Two-Step RT-PCR SuperMix (TransGen Biotech). PCR amplification was performed with reagents from TransGen Biotech. The Human APP cDNA was amplfied using primers as follows: 5'-GACTGACCACTCGACCAGGTTCTG-3' (upstream), 5'-CTTGAAGTTGGATTCTCATACCG-3' (downstream); GAPDH: 5'-TTCAACGGCACAGTCAAGG-3' (upstream), 5'-CACCAGTGGATGCAGGGAT-3' (downstream). Amplification was performed as follows: APP: 35 cycles of 95°C for 30 s, 62°C for 30 s and 72°C for 30 s; GAPDH: 30 cycles of 95°C for 45 s, 58°C for 45 s, and 72°C for 60 s. The PCR products were normalized in relation to standards of GAPDH mRNA.

### Sandwich ELISA

Brain samples from APP/PS1 transgenic mice were homogenized in 5.0 M guanidine buffer (diluted in standard dilution) and centrifuged for 30 min at 4°C. The supernatants were then loaded onto 96-well plates and the level of soluble Aβ1-42 was determined using an ELISA kit (Invitrogen) in accordance with the instructions of the manufacturers. The absorbance was recorded at 450 nm using a 96-well plate reader.

### Statistical analysis

All values are expressed as mean ± standard deviation (SD). Differences between groups were analyzed by Student's *t*-test between STZ-induced insulin deficient diabetic AD model mice and vehicle controls. All data were analyzed using SPSS software, and differences were considered significant at p < 0.05.

## Competing interests

The authors declare that they have no competing interests.

## Authors' contributions

XW contributed to the general design and performed the experiments, and contributed to the statistical data analysis and the writing of the manuscript. WZ performed the statistical analysis. JX contributed to the writing and review of the manuscript. TW contributed to the analysis of the studies. SW participated in its design and coordination. ZW and WT contributed to the overall experimental design, data interpretation and critical manuscript review. All authors read and approved the final manuscript.
